# Oxygen Uptake Efficiency Slope is Strongly Correlated to VO_2peak_ Long-Term After Arterial Switch Operation

**DOI:** 10.1007/s00246-021-02554-9

**Published:** 2021-02-01

**Authors:** Covadonga Terol Espinosa de los Monteros, Roel L. F. Van der Palen, Mark G. Hazekamp, Lukas Rammeloo, Monique R. M. Jongbloed, Nico A. Blom, Arend D. J. Ten Harkel

**Affiliations:** 1 Division of Paediatric Cardiology, Department of Paediatrics, Leiden University Medical Center, Leiden, The Netherlands; 2 Department of Cardiothoracic Surgery, Leiden University Medical Center, Leiden, The Netherlands; 3 Division of Paediatric Cardiology, Department of Paediatrics, Amsterdam University Medical Centre, Amsterdam, The Netherlands; 4 Department of Cardiology, Leiden University Medical Center, Leiden, The Netherlands

**Keywords:** Oxygen uptake efficiency slope, Congenital heart disease, Transposition of the great arteries, TGA, Arterial switch operation, Cardiopulmonary exercise testing

## Abstract

After the arterial switch operation (ASO) for transposition of the great arteries (TGA), many patients have an impaired exercise tolerance. Exercise tolerance is determined with cardiopulmonary exercise testing by peak oxygen uptake (VO_2peak_). Unlike VO_2peak_, the oxygen uptake efficiency slope (OUES) does not require a maximal effort for interpretation. The value of OUES has not been assessed in a large group of patients after ASO. The purpose of this study was to determine OUES and VO_2peak_, evaluate its interrelationship and assess whether exercise tolerance is related to ventricular function after ASO. A cardiopulmonary exercise testing, assessment of physical activity score and transthoracic echocardiography (fractional shortening and left/right ventricular global longitudinal peak strain) were performed to 48 patients after ASO. Median age at follow-up after ASO was 16.0 (IQR 13.0–18.0) years. Shortening fraction was normal (36 ± 6%). Left and right global longitudinal peak strain were reduced: 15.1 ± 2.4% and 19.5 ± 4.5%. This group of patients showed lower values for all cardiopulmonary exercise testing parameters compared to the reference values: mean VO_2peak_% 75% (95% CI 72–77) and mean OUES% 82(95% CI 77–87); without significant differences between subtypes of TGA. A strong-to-excellent correlation between the VO_2peak_ and OUES was found (absolute values: *R* = 0.90, *p* < 0.001; normalized values: *R* = 0.79, *p* < 0.001). No correlation was found between cardiopulmonary exercise testing results and left ventricle function parameters. In conclusion, OUES and VO_2peak_ were lower in patients after ASO compared to reference values but are strongly correlated, making OUES a valuable tool to use in this patient group when maximal effort is not achievable.

## Introduction

After correction with the arterial switch operation (ASO), long-term survival and outcome of patients with transposition of the great arteries (TGA) are usually good. However, residual lesions, including right ventricular outflow tract obstruction, aortic root dilatation, aortic insufficiency and left ventricular dysfunction with or without coronary artery abnormalities can contribute to increased morbidity [1, 2]. Moreover, impaired exercise tolerance has been described in patients after ASO, sometimes already being present at a young age [3–8]. Contributing factors to reduced exercise tolerance have been shown to include chronotropic incompetence, narrowing of the main pulmonary artery with or without pulmonary branch obstruction, coronary abnormalities, ventricular dysfunction and longer follow-up time after ASO [5–10]. To test exercise performance, the gold standard is the cardiopulmonary exercise test with measurement of the maximal oxygen consumption (VO_2peak_) [11], but it requires the capacity to perform maximal exercise for its interpretation. In certain patient groups, e. g. young children, the required maximal exercise during a cardiopulmonary exercise test will often not be reached due to motivational aspects. In addition, patients with mental disability or with certain cardiovascular diseases may have reduced capacity to fulfil the required maximum exercise. These considerations make the use of submaximal exercise parameters such as the ventilatory efficiency (VE/VCO_2_slope) and the oxygen uptake efficiency slope (OUES) potentially valuable [12]. OUES has been investigated in both healthy subjects and patients with congenital heart defects over a wide age range and it was shown to be an objective and effort-independent cardiopulmonary exercise test parameter, strongly correlated to VO_2peak_ [13–16]. However, its value in patients after ASO has not been previously determined. Therefore, the aim of the present study was to correlate OUES as a submaximal exercise parameter to VO_2peak_ as a maximal exercise parameter in a group of patients after ASO. In addition, we studied whether exercise tolerance could be related to ventricular function or right ventricle outflow tract obstruction as assessed by echocardiography.

## Material and Methods

Forty-eight patients with TGA with intact ventricular septum or with ventricular septal defect after ASO were included. Patients with complex TGA including Taussig-Bing anomaly, prior left ventricular outflow tract obstruction or aortic arch obstruction were excluded. A cardiopulmonary exercise test, assessment of physical activity score and a transthoracic echocardiogram were performed.

All patients performed a progressive cardiopulmonary exercise test on an electronically braked cycle ergometer (GE Healthcare eBike Comfort, Freiburg, Germany) up to exhaustion. A facemask (Hans Rudolph, Kansas City, MO, USA) connected to a flowmeter (Triple V volume transducer) and a computerized gas analyser (Jaeger MasterScreen CPX, CareFusion GmbH, Hoechberg, Germany) which calculated breath-by-breath minute ventilation (VE), oxygen uptake (VO_2_), carbon dioxide production (VCO_2_) and respiratory exchange ratio (RER, defined as the ratio VCO_2_/VO_2_) in 10 s intervals were used. Heart rate (HR) was continuously monitored through a twelve-lead electrocardiogram and blood pressure was determined every 2 min by sphygmomanometry. A 3 min warm-up phase (unloaded cycling) was followed by a continuous incremental bicycle protocol with a work rate increment of 10, 15 or 20 W/min depending on the height (< 125 cm, 125–150 cm or > 150 cm) according to Godfrey protocol [17]. The patients had to maintain a pedalling rate between 60 and 80 revolutions/min and were encouraged to perform to exhaustion. The cardiopulmonary exercise test could be terminated by the patient in case of discomfort or by the physician in case of ECG changes, excessive breathing pattern or otherwise. Test with a peak RER (RER_peak_) of ≥ 1.00 were included for analysis.

The RER_peak_ was calculated as the average of 2 highest consecutive achieved RER values in 10 s during peak work rate_._ Peak work rate was defined as the maximum work rate achieved and finished (1 min completed) and the %predicted value was calculated [18, 19]. HR at rest was measured after at least 3 min in a seated position and HR peak was calculated as the highest value achieved during at least 10 s in peak work rate. Then the %predicted value was calculated with the formula [200-age], being abnormal < 85% [11]. HR reserve was defined as HR peak minus HR rest. HR was also recorded at 1 and 2 min after cessation of the cardiopulmonary exercise test (HR01′ and HR02′). HR recovery was calculated as the difference between HR peak and HR01′ and HR peak and HR02′. The relative decrement in HR (HR01% and HR02%) was calculated as (HR recovery/HR reserve) × 100%.

VO_2peak_ (ml/min) was calculated as the average of 2 highest consecutive achieved VO_2_ values in 10 s during WR_peak_. Reference values were used for the interpretation of the results from the exercise tests and for calculation the %predicted value of VO_2peak_ (VO_2peak_%) [20]. A VO_2peak_% value was considered abnormal < 85%. VE/VCO_2_slope is the slope of the linear regression of VE and VCO_2_ during the entire period of the test. The O_2_pulse is the VO_2_ divided by HR and the maximal O_2_pulse (O_2_pulse_max_) was calculated as the average of the highest two consecutive O_2_pulse values during WR_peak_. The data of Ten Harkel et al. [18] was used to calculate the % predicted values of O2 pulse. OUES was calculated by the linear least squares regression of the VO_2_ on the common logarithm of the VE by the equation VO_2_ = alog (VE) + b, where the constant ‘a’ is the regression coefficient OUES [12]. Absolute values, %predicted values and values per body weight were represented. Weight and height were obtained and body surface area (BSA) and body mass index (BMI) were calculated by using the Dubois equation. The %predicted value of OUES (OUES%) was determined using the previous described formulas based on reference normal values adjusted for age and sex [13, 16].

A lifestyle interview was performed to evaluate patients’ weekly exercise behaviour according to the previously described method [21]. In short, patients were queried on their voluntary exercise behaviour (e.g. swimming, fitness, tennis, jogging, soccer) and physical activities related to transportation (cycling, walking) and compulsory physical education classes. Only activities done for at least 6 months and more than 3 months per year were included. Each exercise activity was converted into a metabolic equivalent task (MET) score [22], and a weekly MET score (METhours/week) was calculated (i.e. MET scores multiplied by the duration of activities and summed).

Transthoracic echocardiography was performed using a commercially available system (Vivid-7.0.0, General Electric Vingmed Ultrasound, Horten, Norway) and images were stored in digital format. Off-line analyses were made using EchoPac version 11.1.8 (General Electric Vingmed). Left ventricular (LV) systolic performance was assessed using LV fractional shortening (FS) in M-mode recordings of the parasternal LV long axis view. LV internal diameter at end-diastole (LVIDd) and LV internal diameter at end-systole (LVIDs) were assessed and FS was calculated as follows: ((LVIDd-LVIDs)/LVIDd) × 100%. Left and right ventricular global longitudinal strain (GLS) was obtained from the apical 4-chamber view using speckle-tracking strain analysis as previously described and according to the international guidelines [23, 24]. In patients with a repaired VSD, care was taken to exclude the patch area from the strain analysis.

Tricuspid regurgitation was identified using colour-flow Doppler in the apical 4-chamber view. Estimation of the right ventricular pressure was performed using CW Doppler by placing the ultrasound beam aligned to the tricuspid regurgitation when it was present. To assess the severity of right ventricular outflow tract obstruction, the maximal right ventricular outflow tract velocity with CW Doppler across main pulmonary artery and right and left pulmonary arteries was measured. Stenosis was graded based on the greatest maximal velocity (Vmax): mild, Vmax = 2–3 m/s; moderate, Vmax = 3–4 m/s; or severe, Vmax > 4 m/s.

Data analysis was performed using SPSS Statistics software (v.25.0 IBM SPSS, Chicago, IL). Variables were tested for normal distribution using the Shapiro–Wilk test. Continuous data were expressed as mean ± standard deviation (SD) or as median and inter-quartile range (IQR) where suitable. The paired samples t-test or the Mann–Whitney *U* test, in case of non-normality, were used to assess differences in cardiopulmonary exercise test or echocardiographic parameters between sex and diagnosis (with intact ventricular septum or with ventricular septum defect). The exercise test results were expressed relatively to the reference values, as % of predicted value (100% would mean equal to reference value) and represented as mean with 95% of confidence interval (CI). To test whether the values of patients differed from their reference values, the one sample *t*-test was used. Correlations between the exercise test parameters and the echocardiographic parameters were calculated as Pearson or Spearman correlation coefficient depending on data distribution. Correlations between age and VO_2peak_ and OUES were performed by Pearson correlation as well. *p* values < 0.05 were accepted as statistically significant.

## Results

Table 1 shows the general characteristics of the study group. Forty-eight patients were included, 37 of them (77.1%) had intact ventricular septum and 11 had ventricular septum defect. One-stage ASO was performed in 95.7% of the patients; in two patients a two-stage approach was performed. Median age at ASO was 6 days (IQR 4–9) and the median age at follow-up was 16.0 (IQR 13.0–18.0) years post-ASO.

**Table 1:** Characteristics of study population

	All patients (*n* = 48)
Demographic characteristics	
TGA-IVS	37 (77.1%)
One-stage repair	46 (95.7%)
Age at ASO (days)	6 [4–9]
Age at study (years)	16.0 [13.0–18.0]
Sex, male	36 (75%)
Weight (kg)	62.7 ± 15.4
Height (cm)	171.6 ± 13.9
Body surface area (m^2^)	1.73 ± 0.26
Body mass index (kg/m^2^)	20.6 ± 3.5
Echocardiographic parameters	
Fractional shortening LV (%)	36 ± 6
Global peak strain LV (%)	-15.1 ± 2.4
Global peak strain RV (%)	-19.5 ± 4.5
Max peak flow velocity in pulmonary arteries	2.57 ± 0.66
Right ventricular pressure (mmHg)	31.2 ± 8.4

Data shown as mean ± SD, median [IQR] or number (%)

*LV* left ventricle, *RV *right ventricle

**Fig. 1:**
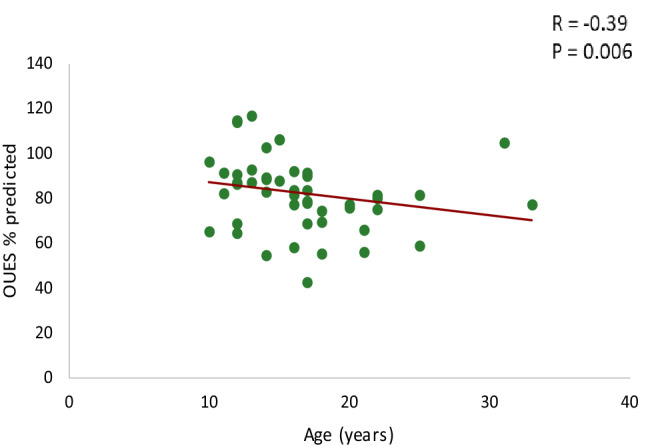
Relationship between age and the percentage of predicted values of OUES. *OUES* oxygen uptake efficiency slope

**Fig. 2:**
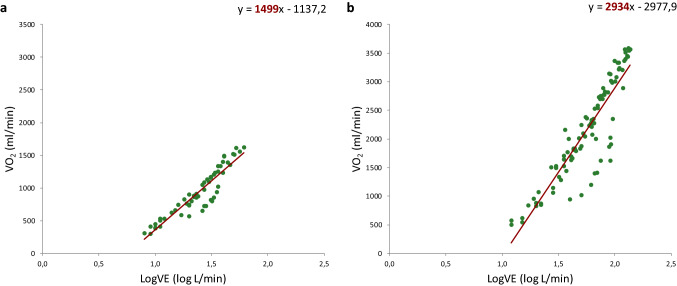
OUES depicted in two patients after arterial switch operation during a cardiopulmonary exercise testing. Panel **a** 12-year-old male patient with OUES = 1499 ml/min (68% of the predicted value). Panel **b** 17-year-old male patient with OUES = 2934 ml/min (90% of the predicted value). A steeper slope represents a more efficient oxygen uptake: the higher the OUES value, the higher the slope is, meaning that a smaller minute ventilation is needed for a determined oxygen uptake. *LogVE* common logarithm of minute ventilation, *VE* minute ventilation, *VO*_*2*_ oxygen uptake

**Fig. 3:**
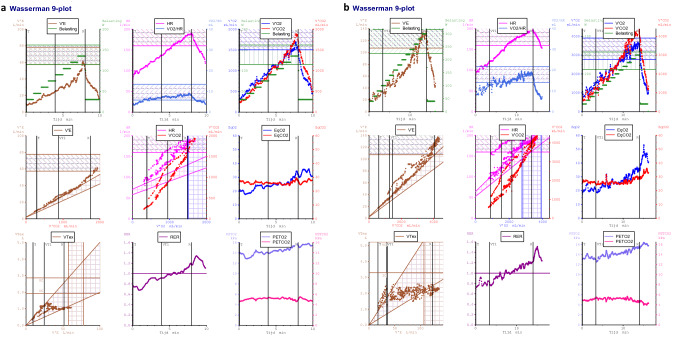
Wasserman 9-plot of the same two patients of Fig. 2. Panel **a** RER_peak_ 1.2, WRp 135 W (92% of the predicted value), HR_peak_ 187 bpm (99% of the predicted value), VO_2peak_ 1616 ml/min (73% of the predicted value), VE 28.03, O_2_pulse_max_ 8.65 ml/bpm (68% of the predicted value). Panel **b** RER_peak_ 1.2, WRp 300 W (122% of the predicted value), HR_peak_ 196 bpm (107% of the predicted value), VO_2peak_ 3564 ml/min (96% of the predicted value), VE 29.7, O_2_pulse_max_ 18.3 ml/bpm (90% of the predicted value). *HR*_*peak*_ maximal heart rate at peak exercise, *O*_*2*_*pulse*_*max*_ maximal O_2_ pulse, *RER*_*peak*_ respiratory exchange ratio at peak exercise, *VO*_*2peak*_ oxygen uptake at peak exercise, *VE* ventilatory efficiency, *WR*_*peak*_ peak work rate

**Fig. 4:**
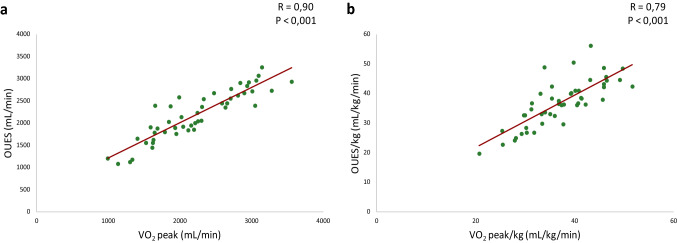
Relationship between OUES and VO_2peak_. Panel **a** shows the relationship of the absolute values and panel **b** the relationship of the values per weight(kg). *OUES* oxygen uptake efficiency slope, *VO*_*2peak*_ oxygen uptake peak

All patients exercised to exhaustion with an RER > 1.0 without any adverse events. Cardiopulmonary exercise test results are depicted in Table 2. TGA patients showed on average lower values for all exercise test parameters compared to reference values from a healthy dataset [13, 16, 20], as reflected by %predicted values: VO_2peak_% = mean 75% (95% CI 72–77), *p* < 0.001; and OUES% = mean 82% (95% CI 77–87), *p* < 0.001. O_2_pulse_max_ was also decreased with a percentage predicted of 79% (*p* < 0.001). No significant differences in cardiopulmonary test parameters were found between patients with intact ventricular septum or with ventricular septum defect. As expected, female patients had significant lower VO_2peak_ and OUES compared to male TGA patients. The %predicted values for male and female of VO_2peak_ (VO_2peak_%: male = mean 74% (95% CI 71–77) vs. female = mean 77% (95% CI 70–84); *p* = 0.358 and OUES (OUES%: male = 84% (95% CI 78–89) vs. female = mean 77% (95% CI 68–87); *p* = 0.505 were not significantly different. There was a significant negative linear relationship between age and OUES% (*R* = -0.39, *p* = 0.006) (Fig. 1) but not between age and VO_2peak_%. The relation between the oxygen uptake and the minute ventilation in two TGA patients with good and bad exercise performance and the Wasserman nine panel plots of this patients are depicted in Figs. 2 and 3 as an illustration. Figure 4 shows an excellent and strong correlation respectively between the VO_2peak_ and OUES, for both the absolute and the normalized data (*R* = 0.90, *p* < 0.001 and *R* = 0.79 *p* < 0.001, respectively).

**Table 2:** Cardiopulmonary exercise test results

	All patients (*n* = 48)	Male (*n* = 36)	Female (*n* = 12)	*p* value
SBP_basal_ (mmHg)	129 ± 15	129 ± 15	126 ± 14	0.512
SBP_peak_ (mmHg)	189 ± 22	189 ± 24	180 ± 21	0.214
RER_peak_	1.19 ± 0.08	1.18 ± 0.08	1.17 ± 0.09	0.738
WR_peak_ (W)	194 ± 52	206 ± 50	156 ± 40	0.004
% predicted	88 (82–94)	88 (81–95)	86 (71–102)	0.787
W/kg	3.14 ± 0.64	3.25 ± 0.65	2.8 ± 0.47	0.022
HR_rest_ (bpm)	82 ± 18	81 ± 17	87 ± 21	0.314
HR_peak_ (bpm)	187 [179–190]	187 [179–190]	187 ± 11	0.939
% predicted	101 (99–103)	101 (98–103)	100 (95–105)	0.853
HR_reserve_ (bpm)	103 ± 18	104 ± 17	99 ± 21	0.899
HR_01%_	31 ± 10	32 ± 11	30 ± 7	0.595
HR_02%_	49 ± 13	49 ± 14	51 ± 11	0.589
VO_2peak_ (ml/min)	2289 ± 599	2424 ± 573	1656 ± 389	** < 0.001**
% predicted	75 (72–77)	74 (71–77)	77 (70–84)	0.358
≤ 84%, *n*(%)	17 (36)	12 (33)	5 (42)	0.601
(ml/kg/min)	37.1 ± 7.2	39.1 ± 6.7	31.2 ± 4.4	** < 0.001**
VE (VE/VCO_2_ slope)	29.2 ± 3.6	28.8 ± 3.1	30.1 ± 4.6	0.273
O_2_pulse_max_ (ml/bpm)	12.3 ± 3.2	13.3 ± 3.0	9.1 ± 2.1	** < 0.001**
% predicted	79 (73–84)	86 (80–91)	58 (49–67)	** < 0.001**
OUES (ml/min/log(L/min))	2191 ± 553	2363 ± 502	1826 [1292–1923]	** < 0.001**
% predicted	82 (77–87)	84 (78–89)	77 (68–87)	0.505
≤ 84%, *n *(%)	27 (56)	21 (58)	6 (50)	0.614
OUES/kg	36.5 ± 7.8	38.9 ± 7.6	32.1 ± 6.7	0.012
OUES/BSA	1283 ± 227	1353 ± 199	1073 ± 171	** < 0.001**

Data shown as mean ± SD, median [IQR] or number (%). The % predicted values were shown as mean (95% CI)

*HR*_*peak*_ maximal heart rate at peak exercise, *HR*_*reserve*_ maximal heart rate-resting heart rate, *HR*_*rest*_ resting heart rate, *HR*_*01%*_ percentage heart rate recovery at 1 min, *HR*_*02%*_ percentage heart rate recovery at 2 min, *O*_*2*_*pulse*_*max*_ maximal O_2_ pulse, *OUES* oxygen uptake efficiency slope, *RER*_*peak*_ respiratory exchange ratio at peak exercise, SBP_basal_ systolic blood pressure at rest, *SBP*_*peak*_ systolic blood pressure at peak exercise, *VO*_*2peak*_ oxygen uptake at peak exercise, *VE* ventilatory efficiency; *WR*_*peak*_ peak work rate

Echocardiographic results are presented as well in Table 1. All patients were in sinus rhythm. LV systolic function represented by the FS was on average 36% ± 6 and above 30% in 94% of all TGA patients. LV and RV GLS were − 15.1% ± 2.4 and − 19.5% ± 4.5 respectively. There was no significant difference in the GLS between patients with IVS and VSD (IVS: − 14.3 ± 2.4 vs VSD: − 13.6 ± 3.9, *p* 0.45). Stenosis in pulmonary arteries was present in 83.3% of patients (64.6% mild and 18.8% moderate stenosis). No correlation was found between exercise test results (VO_2peak_ and OUES) and ventricular function parameters (FS, LV and RV GLS), the maximal CW Doppler measured in pulmonary arteries or the maximal gradient of the tricuspid regurgitation. Moreover, there were no significant differences between the 24 patients with the highest and the 24 patients with the lowest VO_2peak/kg_ values in the Vmax across de RVOT (2.67 m/s vs 2.48 m/s, *p* = 0.312).

Average weekly MET score was 52.5 ± 26.2, without significant differences between males and females (male = 54.6 ± 27.8 vs. female = 47.3 ± 23.3, *p* = 0.581). One patient scored < 21 METhour/week. Weekly MET score showed a significant moderate correlation with the VO_2peak_% (*R* = 0.55, *p* = 0.009).

## Discussion

The results of this study demonstrate that patients post-ASO have diminished exercise capacity, depicted in both lower VO_2peak_ and OUES. OUES and VO_2peak_ showed an excellent correlation which supports the value and importance of OUES as an effort independent cardiopulmonary exercise test parameter. No correlation was found between VO_2peak_ or OUES and ventricular function parameters (FS, LV and RV GLS) or the maximal CW Doppler gradient measured in pulmonary arteries.

As previously mentioned in certain patients groups such as young children or older patients with multiple morbidities maximal exercise during cardiopulmonary exercise test will often not be reached. Therefore, it would be of importance if exercise parameters during submaximal exercise would give the same information as the gold standard parameter VO_2peak_. In previous studies it has been shown that OUES, a parameter obtained during submaximal exercise, is an objective effort-independent parameter to evaluate cardiopulmonary fitness [12, 14, 15, 25, 26]. To our knowledge OUES has not been previously evaluated in a group of children with congenital heart defects and in particular children with TGA after ASO. In the present study we found an excellent correlation between OUES and VO_2peak_ and between indexed to weight OUES and VO_2peak_ in TGA patients after ASO. These findings make it possible to perform submaximal exercise and gain similar information as VO_2peak_ in conditions where maximal exercise is less appropriate as in young children or during e.g. a postoperative course after reoperations.

A reduced exercise performance was present in our paediatric population of TGA patients, which confirms the results of previous studies [3, 5–8]. Both the submaximal value of OUES as well as the VO_2peak_ were reduced as compared to a reference population. Cardiopulmonary exercise testing with the use of continuous measurement of HR, VO_2_ and VCO_2_ assess the integrity of the pulmonary system, cardiovascular system, autonomic nervous system and peripheral muscles. In patients with congenital heart disease all these factors may play a role in the presence of reduced exercise capacity. The importance of an intact autonomic nervous system in congenital heart defects patients is revealed by the fact that a reduction in parasympathetic nervous activity may contribute to increased mortality. HR recovery after cessation of exercise is mainly driven by the parasympathetic system. In the present study HR recovery after 1 min was 31%, which corresponds to the value obtained in healthy female subjects in other studies, but was a bit lower in males (31% vs 35%) [18, 27]. Sympathetic denervation after ASO has been postulated as one of the possible causes of reduced chronotropic competence in TGA patients [28]. However, in the present study we did not find a reduced maximal HR, which correlates to several studies [3, 6], although some other studies did find a reduced maximal heart rate [7, 8]. The % predicted value of O_2_pulse_max_ was reduced in our patients and this could reflect a reduced stroke volume at peak exercise [29].

Ventilatory efficiency as assessed by the VE/VCO_2_ slope is reduced in many congenital heart defects patients. This measure is highly related to long-term outcome. In our study the VE/VCO_2_slope of the TGA patients was similar to the values of healthy children previously published (i.e. VE/VCO_2_slope of 30) [13, 18].

Systolic ventricular function is an important factor in exercise tolerance. Although we found a normal FS in this study, both LV and RV global longitudinal peak strain were reduced [24, 30]. Reduced LV longitudinal strain has also been found previously after ASO [31, 32]. A main residual lesion in TGA patients is often the presence of pulmonary stenosis, either of the main pulmonary artery or the pulmonary branches. In many adult patients after ASO, a decreased area in the main pulmonary artery and/or pulmonary branches has been reported to be correlated toVO_2peak_ [9]. In addition, an abnormal (right/left) pulmonary blood flow distribution has also been associated with a decrease exercise capacity [9, 10], and other show that an increased right ventricle outflow tract velocity was correlated to a reduced VO_2peak_ [5]. Although the presence of mild to moderate stenosis in pulmonary branches was common in this study, a correlation with lower VO_2peak_ or OUES could not be detected.

It is as yet unclear if the exercise tolerance in TGA patients will decrease over time. In the present study we found a negative correlation between %OUES and age. However, another study did not find any correlation between age and exercise capacity [5]. Many studies have investigated the role of daily exercise behaviour and the effect of training in congenital heart defects patients. Also, TGA patients have found not to achieve the physical activity levels as recommended in guidelines [33], although other studies found similar levels of physical activity in TGA patients and healthy control subjects [8]. In the present study the weekly MET level was comparable to normal values. However, we observed lower weekly exercise behaviour significantly correlating to diminished exercise capacity. In a large review it was concluded that in children and young adults with congenital heart defects exercise is safe and an improvement of fitness after a physical exercise training programme can be obtained [34]. The findings of the correlation between weekly exercise behaviour and exercise capacity in this study suggests that this effect of training might be valuable in TGA patients as well, and performing regular physical activity in these patients should be stimulated.

Our study has several limitations. The correlation between age and exercise capacity may as well reflect the surgical era, notwithstanding operative techniques have not changed significantly during this period. The measurement of weekly exercise behaviour has been performed by questionnaires and therefore remain a subjective parameter. Measurement by accelerometer or continuous HR monitoring could have been more exact. And finally, although this paper presents one of the larger studies about TGA and exercise performance the power might be too low to find more subtle correlations as with e.g. systolic ventricular function.

In conclusion, we found a good correlation between OUES and VO_2peak_ in a group of TGA patients after ASO, making OUES a valuable tool to use in this patient group when maximal effort is not possible. Still, longer-term follow-up studies are needed to determine whether OUES is a reliable parameter for determining exercise capacity in this group of patients and whether the correlation with the VO_2peak_ remains consistent. The lower exercise performance in our group as assessed by both VO_2peak_ and OUES might be multifactorial, including decreased parasympathetic activity, while other potential factors as pulmonary stenosis and systolic ventricular function may contribute, although not significantly correlated in the present study. Furthermore, deterioration in exercise capacity over age may play a role, while increasing daily exercise performance may contribute to stabilizing exercise capacity.
